# Characterization 0.1 wt.% Nanomaterial/Photopolymer Composites with Poor Nanomaterial Dispersion: Viscosity, Cure Depth and Dielectric Properties

**DOI:** 10.3390/polym13223948

**Published:** 2021-11-15

**Authors:** Rytis Mitkus, Marlitt Scharnofske, Michael Sinapius

**Affiliations:** Technische Universität Braunschweig, Institute of Mechanics and Adaptronics, Langer Kamp 6, 38106 Braunschweig, Germany; marlittscharnofske@gmx.de (M.S.); m.sinapius@tu-braunschweig.de (M.S.)

**Keywords:** photopolymer resin, conductive nanomaterial, multi-walled carbon nanotubes, graphene nanoplatelets, carbon black, 3D printing, viscosity, cure depth, dielectric properties

## Abstract

Notably, 3D printing techniques such as digital light processing (DLP) have the potential for the cost-effective and flexible production of polymer-based piezoelectric composites. To improve their properties, conductive nanomaterials can be added to the photopolymer to increase their dielectric properties. In this study, the microstructure, viscosity, cure depth, and dielectric properties of ultraviolet (UV) light curable 0.1 wt.% nanomaterial/photopolymer composites are investigated. The composites with multi-walled carbon nanotubes (MWCNTs), graphene nanoplatelets (GNPs), and carbon black (CB) are pre-dispersed in different solvents (acetone, isopropyl alcohol, and ethanol) before adding photopolymer and continuing dispersion. For all prepared suspensions, a reduction in viscosity is observed, which is favorable for 3D printing. In contrast, the addition of 0.1 wt.% nanomaterials, even with poor dispersion, leads to curing depth reduction up to 90% compared to pristine photopolymer, where the nanomaterial dispersion is identified as a contributing factor. The formulation of MWCNTs dispersed in ethanol is found to be the most promising for increasing the dielectric properties. The post-curing of all composites leads to charge immobility, resulting in decreased relative permittivity.

## 1. Introduction

The research on thin and flexible two-component 0-3 piezoelectric composites lasts for a few decades. The advantages of the addition of conductive carbon-based nanomaterials to improve piezoelectric properties of so-called 0-0-3 piezoelectric composites were reported by researchers [1,2,3,4,5,6]. The improvement of piezoelectric properties in such composites is achieved by increasing the dielectric properties of the polymer matrix by adding conductive nanomaterials to the matrix. Increased dielectric properties allow one to achieve a higher degree of polarization of piezoelectric particles inside the matrix, and thus higher piezoelectric output [2,3,5,6,7,8].

Recently, the possibility of using stereolithography (SLA) or digital light processing (DLP)-type 3D printing processes to print non-standard geometry 0-3 piezoelectric composite sensors was reported by researchers [9,10]. The SLA/DLP 3D printing technique uses ultraviolet (UV) light (either laser or LED UV light source) to solidify photopolymer, layer after layer, to obtain the final part. Moreover, 3D printable materials allow achieving non-standard geometries of piezoelectric composite sensors that, in turn, can improve signal acquisition [11,12,13], reduce sensor mass, and reduce signal reflection in structural health monitoring (SHM) applications [14]. We believe that it is possible to increase the piezoelectric properties of 3D printable two-component piezoelectric composites by adding the third phase—conductive, carbon-based nanofillers to obtain high-performance, 3D printable 0-0-3 piezoelectric composites. As a first step to achieving high-performance 3D printable piezoelectric composites, the influence of conductive nanofillers on dielectric properties, cure behavior, and viscosity of 0-3 nanofiller/photopolymer composites is studied here.

The material suitability for 3D printing is defined by a small or no decrease in the curing depth of a photopolymer, and a low viscosity of nanofiller/photopolymer suspensions before printing. The low viscosity is important for the further addition of piezoelectric ceramic particles envisioned in the future. It is expected that the addition of small amounts of nanofillers will only slightly increase the viscosity. Curing depth defines how a thick layer of the material can be formed over time with a specific UV light dose. It directly influences the printing resolution and printable layer thickness [15]. Furthermore, by decreasing the overall degree of the cure of the material, lower mechanical and electrical properties of the nanofiller/photopolymer composite can be expected. After solidification of the photopolymer, the final properties of the material are not achieved yet, and post-curing in additional UV light and heat must be conducted to achieve the final electrical and mechanical properties of the material. The influence of curing degree on dielectric properties currently cannot be quantified. The dielectric properties of the composites manufactured in this study are measured on just cured nanomaterial/photopolymer specimens, and on post-cured specimens.

One of the most complex tasks is the dispersion of nanomaterials into a polymer, where the methods and parameters used vary greatly in the literature [1,2,3,4,5,6]. In this study, only 0.1 wt.% of multi-walled carbon nanotubes (MWCNTs), graphene nanoplatelets (GNPs), and carbon black (CB) is used. Every nanomaterial has different particle geometry and size. All nanomaterials investigated here are dispersed in a photopolymer, with the help of solvent and an ultrasonic bath. Three different solvents are investigated: acetone, isopropyl alcohol, and ethanol, yet ethanol was discarded from further experiments after bubble formation in the initial experiments.

The main aim of this study is twofold: find which nanomaterial produces the highest dielectric properties, the lowest influence on cure depth, and a low change in viscosity. Secondly, important questions to ask are which dispersant helps to achieve good dispersion of nanomaterials, and how the dispersant influences the above-mentioned properties. Furthermore, additional measurements of dielectric properties are conducted after post-curing of the specimens to understand the post-curing influence on dielectric properties.

### State-of-the-Art

In the past, it has been shown that carbon-based nanomaterials such as carbon nanotubes (CNTs), GNPs, and CB can improve the electrical and mechanical properties of polymer-based composites [16,17,18,19,20]. The highest improvement in dielectric properties is achieved close to the so-called percolation threshold (hence p_c_) [20,21]. The percolation threshold defines the amount of conductive nanofiller required to achieve conductivity [22].

A weight fraction of only 0.1 wt.% MWCNTs can be sufficient for reaching p_c_ and a high increase in the electrical conductivity of SLA/DLP printed composites [23,24]. Gonzalez et al. [23] reported an increase from 2·10^−9^ S/cm (0 wt.%) to 1·10^−7^ S/cm (0.1 wt.%). In contrast, Cortés [25] achieved a conductivity in the range of 1·10^−8^ S/cm at the p_c_, with the addition of only 0.05 wt.% MWCNT to a photopolymer. In the past, it has been shown that, for GNP/epoxy and CB/epoxy composites, higher weight fractions in the range from 0.5 wt.% up to 20 wt.% can be necessary to achieve an appropriate improvement in electrical properties [19,20,21,26,27]. Weight fractions required to achieve higher conductivity depend highly on filler geometry [21]. Due to the correlation of viscosity with nanomaterial content, the printability of highly loaded nanomaterial/resin mixtures can be limited. With a maximum printable GNP content of 2.5 wt.%, the composites of León and Molina did not reach p_c_ [28]. However, the electrical conductivity of about 1·10^−6^ S/cm was measured, which is in good agreement with the work of Joo [29].

From the literature, it is clear that even at low filler content, significant improvements to dielectric properties up to the percolation threshold can be achieved. On the other hand, higher filler content has a negative influence on curing depth. Therefore, this study is limited to a filler content of 0.1 wt.%, only for all material compositions.

The uniform dispersion of nanomaterial and defect-free microstructure of the composites is fundamental for optimum performance [30]. Three-roll-milling, also known as calendaring, or ultrasonication, is therefore a common technique to disperse nanomaterials into viscous polymers. Since photosensitive resins have relatively low viscosity, the re-agglomeration of nanofillers can be observed in the absence of calendaring shear forces [25]. In addition, the reduction of agglomeration is limited by the gap distance of the rolls [31]. Sonication is a suitable method for the deagglomeration of nanomaterials as a pre-treatment or material mixing. In contrast to three-roll-milling, ultrasonic sonication usually requires the use of a solvent. Therefore, solvents like isopropyl alcohol, ethanol, Dimethylformamide (DMF), and Triton x-100 are used in the literature as dispersants [23,24,32]. The choice of solvent has to be adapted to the nanomaterial and polymer to obtain a uniform dispersion without damaging the materials. Other factors are the parameters of the sonication devices and the concentration of the solvent [33,34,35,36]. Moreover, nanomaterial/solvent concentration in the literature varies widely as mentioned by Wei [37]. A low nanomaterial/solvent concentration may facilitate dispersion but also results in prolonged evaporation of the solvent. This can lead to nanomaterial re-agglomeration [37]. Furthermore, some solvents lead to the exfoliation of the nanomaterial [6,33]. Possible chemical reactions have to be avoided [36]. In addition, residual solvents can affect further processing steps [31,38].

## 2. Materials and Methods

In this study, 0.1 wt.% of the respective nanomaterials are dispersed in a commercially available photopolymer matrix, which is normally used in SLA-type 3D printing processes. The photopolymer used is an acrylate-based “High-Temperature V2” photopolymer resin (Formlabs, Somerville, Massachusetts, United States) [39]. This material can achieve high-temperature resistance, high mechanical properties, low elongation, and has brittle behavior when fully post-cured. The material is selected based on our earlier experiments with photopolymer suitability for ceramic/photopolymer composites [40].

### 2.1. Nanomaterials

**Figure 1:**
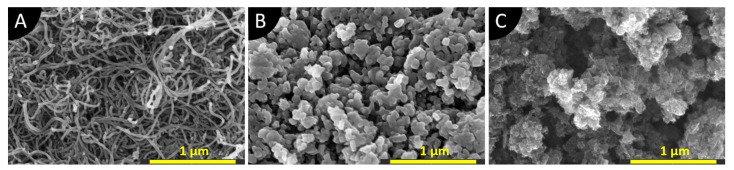
SEM images of as-received nanomaterials. Magnification 150,000×: (**A**) MWCNT-COOH; (**B**) Printex XE2 B Beads® carbon black; (**C**) Graphene nanoplatelets (CP-0081-HP-0100).

Three types of nanomaterials are used in this study. MWCNTs are functionalized with carboxylic functional groups (COOH-) by the manufacturer, and are obtained from FutureCarbon GmbH, Bayreuth, Germany. Graphene nanoplatelets (GNPs) (CP-0081, particle size 2 nm, specific surface area 750 m^2^/g, purity 99.5%) are purchased from Ionic Liquids Technologies GmbH, Heilbronn, Germany. The carbon black (CB) being used is the highly conductive Printex XE2 B Beads^®^ (particle size 30 nm) from ORION Engineered Carbons GmbH, Cologne, Germany. Figure 1 shows the scanning electron microscopy (SEM) images (made with Helios G4 CX DualBeam™, Thermo Fisher Scientific, Waltham, MA, United States) of the nanomaterials used in this study. Nanomaterials were glued on the metal holders with the help of conductive silver ink. The specimens were sputtered with a 4 nm layer of platinum to increase the quality of the SEM images. The SEM images in Figure 1A (MWCNTs) and Figure 1B (CB) show similarities to the morphology of these materials described in the literature [41,42]. In contrast to the elongated carbon nanotubes, the particles of CB are round. Both materials form the agglomerations of diameters up to 50 µm. Figure 1C shows an agglomeration of GNPs of 1 µm in size, but without the flaky structure which is known for GNP from the literature [37,43]. Instead, the morphology appears similar to CB. As reported by Müller [26], the morphology of GNPs can vary greatly, and thus lead to different composite properties for the same filler content.

They classified the nanoplatelets by lamellar, fragmented, and bulk [26]. Looking at Figure 1C, the GNPs used in this study are considered as fragmented. Based on the SEM images, it is expected that every material will exhibit different dispersion characteristics, as well as different influences on suspension viscosity and achievable cure depth. All materials have been used as received without further modifications.

### 2.2. Preparation of 3D Printable Suspensions

**Figure 2:**
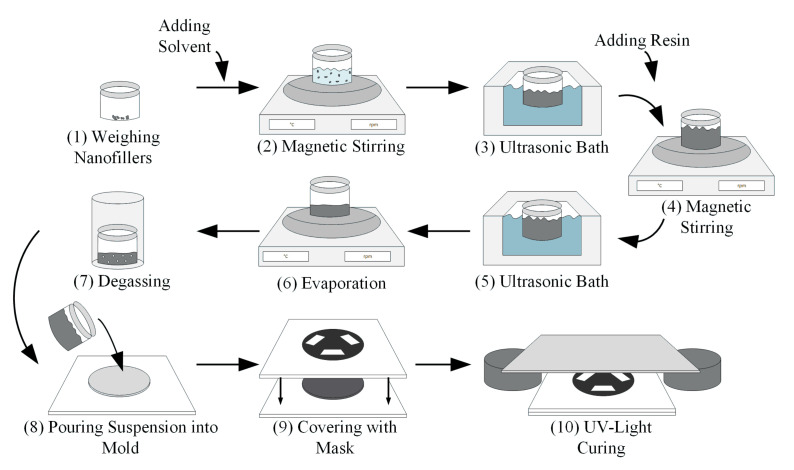
Schematic process diagram of suspension preparation and 0-3 nanocomposite manufacturing.

In literature, highly varying methods to disperse nanomaterials are used. In this study, both ultrasonic bath and magnetic stirrer are used to disperse the nanomaterial in the solvent, as well as mix the resulting dispersion with a photopolymer. The 3D printable suspension preparation procedure is graphically illustrated in Figure 2.

Firstly, the nanomaterial is pre-dispersed in the solvent at a concentration of 0.2 mg/mL. Pre-dispersion is done by stirring the materials with a magnetic stirrer for 30 min at 500 rpm. Subsequently, the dispersion is sonicated by an ultrasonic bath (Palssonic PTIC-10-MF, ALLPAX GmbH & Co. KG, Papenburg, Germany) with a frequency and power of 40 kHz and 300 W, respectively. Pulsed ultrasonication is used to slightly reduce material heating: 0.8-s ultrasonication, 0.2-s pause. The plastic cup with dispersion is placed inside the ultrasonic bath filled with water. Before every use of an ultrasonic bath, the water is changed to keep it at room temperature. The sonication is carried out for 30 min. Afterward, the photopolymer is added, and dispersion is stirred for 15 min. Lastly, ultrasonication for 15 min (0.8-s ultrasonication, 0.2-s pause) is applied.

After the last ultrasonication step, the dispersion is filled in a glass jar and the solvent is evaporated in a few hours, while stirring and heating up to 50 °C. The evaporation is controlled by weighing the mixture until the honey-like suspension is achieved. As a final step before material use for specimen fabrication and characterization, the material is degassed at room temperature for several hours to eliminate any entrapped air.

In this study, all suspensions are prepared with the same parameters. The processing is carried out as precisely as possible.

### 2.3. Manufacturing of Nanocomposites

**Figure 3:**
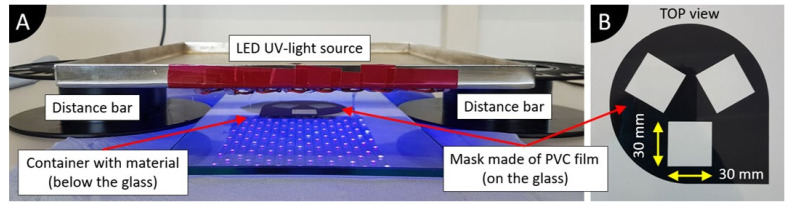
Curing setup used to manufacture rectangular specimens and to measure cure depth: (**A**) Curing setup; (**B**) Mask used to manufacture rectangular sensors.

For SEM and dielectric measurements, solid specimens are required. A simulated DLP-type 3D printing process allows one to cure the suspensions and achieve required geometry specimens. Single-layer specimens are made in this study. Figure 3A shows the setup of the simulated 3D printing process and Figure 3B shows the mask used to manufacture rectangular specimens. The mask is a sticker made of PVC film (Oracal 751C, thickness 60 μm) [44] which is cut with a plotter and is glued on the glass. The same sticker is used throughout the study because it can be easily cleaned with isopropyl alcohol.

The suspension is filled in a 3D-printed plastic container with a height of 1 mm below the glass. Afterwards, the glass with a mask is placed carefully on top, so that suspension would touch the glass surface completely without any bubbles. Excess material flows through the sides of the container. Then, the UV light is applied for up to 8 min to achieve specimens with a thickness of around 0.2 mm. The exact cure time varied between suspensions (from 5 to 8 min), depending on the results of the cure depth experiment.

A customized LED UV light source is used, made of LED light strips (5 m in length with 150 LEDs, 24 W, a wavelength of 395 nm, UV-30, Renkforce, Conrad Electronic SE, Hirschau, Germany). The area of light is 0.05 m^2^; therefore, the overall power of our setup is around 48 mW/cm^2^. While the ideal curing wavelength for the photopolymers used is 405 nm, it is expected that a 10 nm deviation will not have a significant influence on the polymerization process, mechanical and electrical properties [45]. The distance of 5 cm between the top of the glass and the UV light source is set by two plastic blocks placed next to the glass plate.

### 2.4. Measurement of Cure Depth

**Figure 4:**
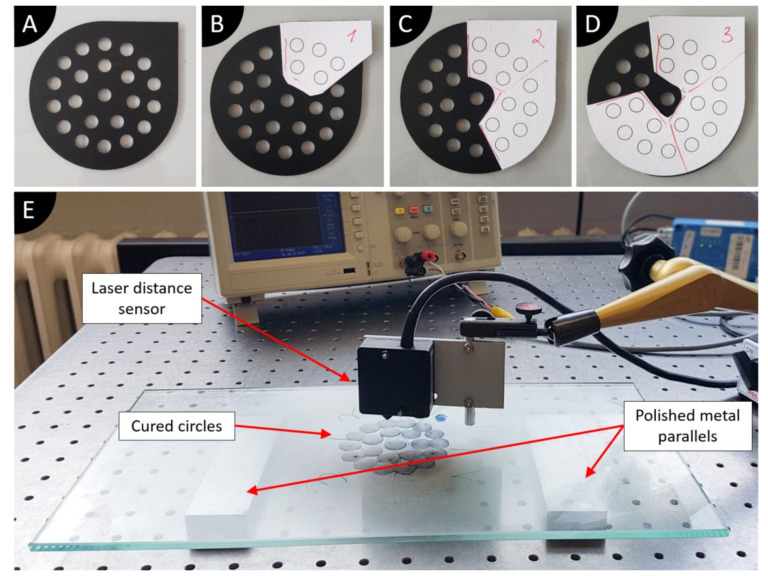
Masks used for cure depth specimen preparation and cure depth measurement setup: (**A**) *t* = 0, no additional mask; (**B**) *t* = 2 min, mask number 1; (**C**) *t* = 4 min, mask number 2; (**D**) *t* = 6 min, mask number 3; (**E**) Cure depth measuring setup.

To measure the cure depth of the suspensions over time, the thickness of solidified material after each 2 min is measured up to the maximum of 8 min cure time. To obtain solid specimens cured with varying exposure times, the same setup as shown in Figure 3A is used, however, a different mask is applied to the glass. Figure 4A shows the mask used for cure depth measurement. The mask is designed in such a way that respective holes could be covered after a certain time with a second mask made of thick paper (card stock) (see Figure 4B–D). Respective holes of the mask are covered after (B) 2, (C) 4, and (D) 6 min of exposure, leading to a total exposure of 8 min for some holes. This cure depth measuring technique is based on a method proposed by Bennett [46].

After the cure of suspension with different exposure times, specimens with a diameter of >8 mm and varying height are formed on the glass. To measure the cure depth, glass is placed on two metal parallels, and all setup is constructed on an air-supported polished table. Two metal parallels allow sliding the glass around as needed to reach every solidified point, and keep the laser distance sensor untouched during measurements. Since the laser cannot directly measure the distance to the glass, a small blue PVC film sticker with a thickness of 90 µm is placed on the glass and serves as a reference point. Because cured suspensions are all translucent, the laser cannot accurately measure the distance to it. Therefore, white pigment (Varybond NDT3 developer, ITW LLC & Co. KG, Mühlacker, Germany) is sprayed onto the cured specimens to create a uniform matte surface. The cure depth of the suspensions is measured with a laser distance sensor (M7L/2, measuring range 2 mm, output ±10 V, wenglorMEL GmbH, Eching, Germany) and an oscilloscope (Tektronix TDS 2002C, Tektronix, Beaverton, Oregon, United State). Firstly, the reference voltages of the blue sticker (cleaned sticker) and white powder are determined. The thickness of the cured samples is determined by pointing the sensor to the highest point of each circle, usually in the center. For every cure time of interest, 5–6 holes are measured, and their average thickness is recorded.

### 2.5. Measurement of Viscosity

Viscosities of all suspensions are measured at room temperature with a parallel plate Advanced Rheometer Gemini 200 h nano (Anton Paar GmbH, Graz, Austria). The plate diameter is 40 mm, and the gap is set to 1 mm. Viscosity is measured at logarithmic shear rate values, between 1 s^−1^ to 30 s^−1^, because non-Newtonian behavior of similar suspensions is observed in earlier experiments [40].

### 2.6. Measurement of Dielectric Properties

Dielectric measurements in this study include measurement of relative permittivity ε_r_ and dissipation factor tan (δ). Firstly, dielectric properties are measured on the cured specimens, which are made as described in Section 3.3. The electrodes of 100 nm, made of gold, were sputtered, leaving about 1 mm around the edges uncovered by gold. Relative permittivity ε_r_ is calculated by measuring the capacitance of specimens with electrodes at different frequencies with LCR meter (LCR-300, Voltrcraft) using Equation (1):

(1) $$\varepsilon_{r}=\frac{C\times d}{\varepsilon_{0}\times A}$$

where *C*—capacitance of the specimen at a respective frequency (F), *d*—average thickness of the specimen (m), *ε*_0_—permittivity of a vacuum, constant (8.84 × 10–12 F/m), *A*—overlapping electrode area of the specimen (m^2^). The relative permittivity and dissipation factor tan (δ) are measured at frequencies: 100 Hz, 1 kHz, 10 kHz, and 100 kHz.

## 3. Results and Discussion

In the results presented below, letters “MWF”, “GNP”, and “CB” represent COOH functionalized MWCNTs, graphene nanoplatelets, and carbon black, respectively. The last letter in the legends indicates the dispersant used, where: A—Acetone; E—Ethanol; I—Isopropyl alcohol. As an example, a composite containing 0.1 wt.% of CB, which is dispersed with acetone, is labeled CBA. “V2” represents pristine photopolymer resin.

**Figure 5:**
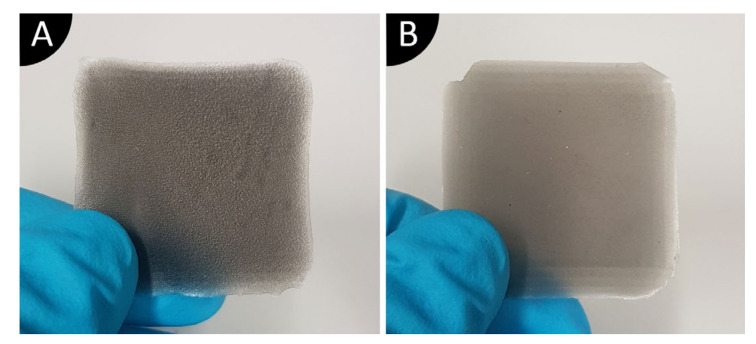
The difference in the top surface quality of cured specimens: (**A**) MWFE (rough top surface); (**B**) MWFI (smooth top surface).

While pouring MWFE suspension into the curing mold, the emergence of numerous small bubbles was observed. After curing, the MWFE samples show significant differences in surface structure and thickness compared to the specimens, whose suspensions are made with other solvents. Figure 5 shows the differences of top surface between MWFE and MWFI specimens after curing with UV light, where the rough surface of MWFE can be seen in Figure 5A. MWFE specimens, which are also thinner than MWFI.

Based only on these observations, it is assumed that ethanol might damage the photopolymer, and it is not a suitable solvent for the 0-3 composites. Furthermore, a rough surface increases electrode area on one side, and dielectric measurements would not be comparable to specimens that have a smooth surface. Ethanol is excluded from use with other nanomaterials in this study.

### 3.1. Microstructure of Nanocomposites

As a first step, the dispersion and distribution of the nanofillers in the composites are investigated. Solidified 0-3 composites are broken, and the cross-sections are inspected with SEM. Cross-sections of broken specimens are sputtered with 4 nm thickness platinum for higher image quality. The results help to assess the effectiveness of the dispersion process and the suitability of the solvents. Irregularities, such as filler agglomerations or voids, are highly undesirable.

**Figure 6:**
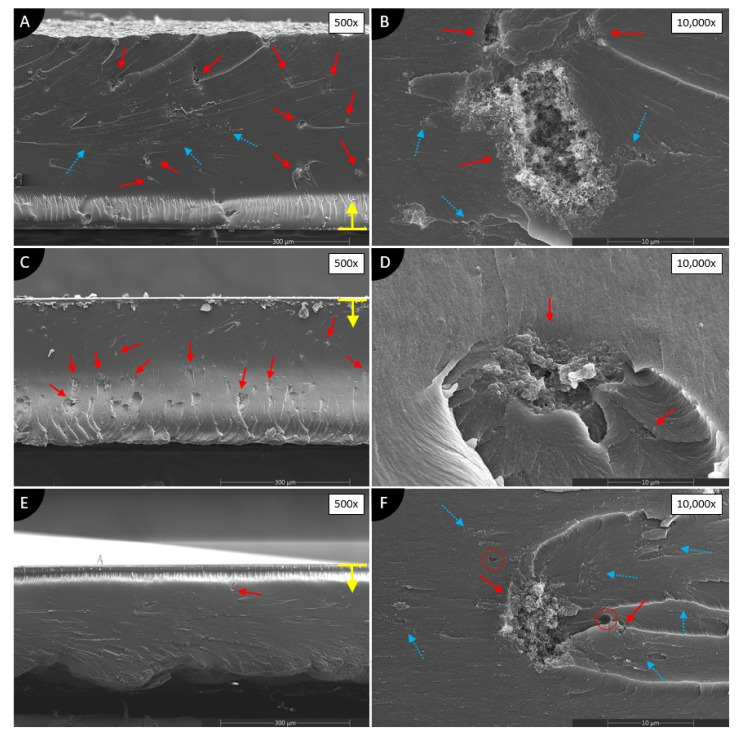
SEM images of cross-sections of 0.1 wt.% MWCNTs/photopolymer composites dispersed in (**A**,**B**) acetone; (**C**,**D**) isopropanol; (**E**,**F**) ethanol. Red, solid line arrows show agglomerations. Blue, dotted arrows show clusters of average dispersion. Red circles define voids. A yellow arrow with a line defines the direction of UV light and the surface of the composite on the glass during manufacturing.

Figure 6 shows SEM images of nanocomposites made of MWCNTs dispersed in photopolymer using different solvents. The side of the composite, from which it began to solidify, is indicated by yellow arrows with a line in SEM images.

MWFA (Figure 6A,B) shows many huge agglomerations of MWCNTs, and at the same time, a few clusters with some average dispersion can be observed. On the other hand, MWFI (Figure 6C,D) shows only many huge agglomerations. No voids are visible in either type of specimens.

During the manufacturing of the specimens, MWFE suspension produced a very rough surface (see Figure 5A). SEM images at low magnification of MWFE (Figure 6E,F) show almost no major agglomerations of MWCNTs. At higher magnification, unfortunately, minor small agglomerations are visible, but overall, the best dispersion of this group of specimens is observed. However, the dispersion forms clusters of average dispersion (see Figure 6F, blue dotted arrows) and it does not spread evenly over the cross-section. Furthermore, a few voids of unknown origin are found in the cross-section (shown by circles in Figure 6F). None of the solvents used produced proper dispersion of the MWCNTs, but MWFE shows the best results.

**Figure 7:**
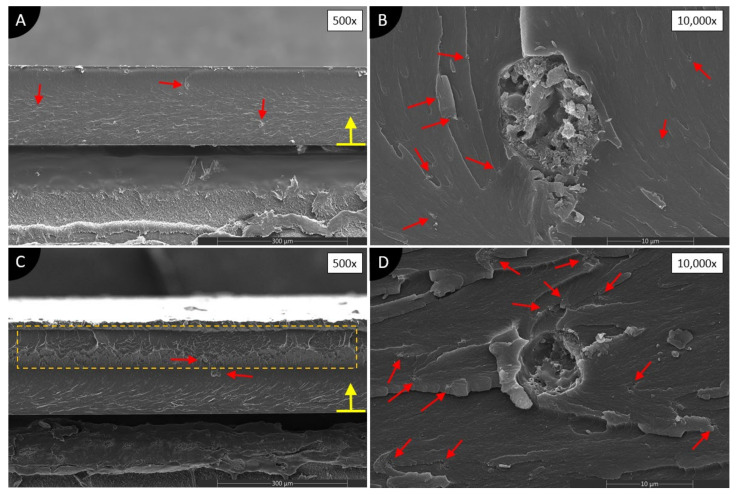
SEM images of cross-sections of 0.1 wt.% CB/photopolymer composites dispersed in (**A**,**B**) acetone; (**C**,**D**) isopropanol. Red, solid line arrows show agglomerations. Orange, dashed rectangular shape depicts an unknown change of the photopolymer. A yellow arrow with a line defines the direction of UV light and the surface of the composite on the glass during manufacturing.

Figure 7 shows composites made with CB and two different solvents. The results of both composite types are very similar, independent of the solvent used. On low magnification (Figure 7A,C), only a few big agglomerations of CB are visible, which are shown with red arrows. Higher magnifications (Figure 7B,D) reveal many small agglomerations around bigger agglomerations. Small agglomerations are randomly scattered and do not cover the whole cross-section area. Big agglomerations, with a size up to 10 μm in diameter, consists of CB, air, and unsolidified photopolymer. Most likely, these agglomerations were filled with a solvent that evaporated during or after solidification. Meanwhile, CBA (Figure 7A) shows quite a smooth surface in a cross-section, CBI, on the other hand, has an interesting layer within the cross-section, marked in an orange, dashed rectangle (see Figure 7C). It looks like a photopolymer in this zone, just partially solidified, and produced gel-like material. Theoretically, this should indicate the better dispersion of nanomaterials, because of reduced curing power throughout the cross-section area. However, it is not clear what exactly that is. Overall, the poor dispersion of CB is achieved.

**Figure 8:**
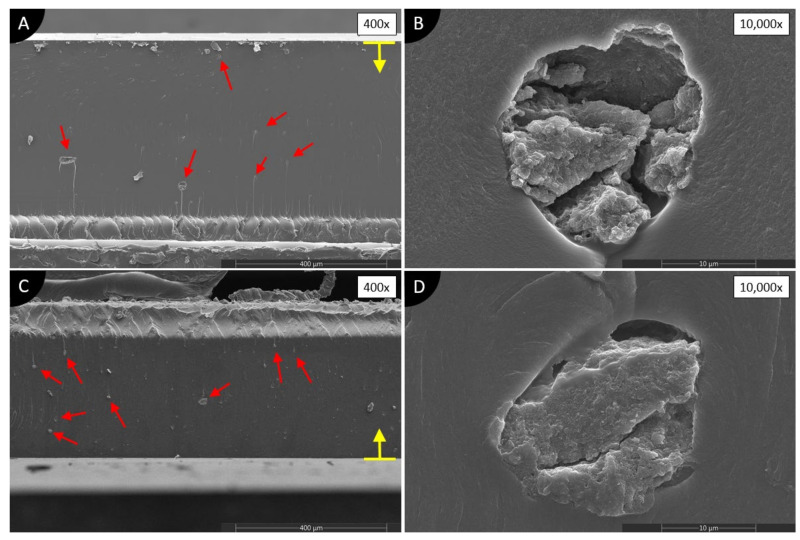
SEM images of cross-sections of 0.1 wt.% GNPs/photopolymer composites dispersed in (**A**,**B**) acetone; (**C**,**D**) isopropanol. Red, solid line arrows show agglomerations. A yellow arrow with a line defines the direction of UV light and the surface of the composite on the glass during manufacturing.

Figure 8 shows the composites made with GNPs and two different solvents. The results of both composites again are very similar, independent of the solvent used. On low magnification (Figure 8A,C), many agglomerations of GNPs are visible, especially in GNPI, but overall, the cross-sections look very smooth and clean in contrast to CB and MWCNT composites. This might indicate the low blocking of UV light, which is desirable. At higher magnitudes (Figure 8B,D), no small agglomerations are visible, as in the case with CB composites. Huge agglomerations consist of agglomerated GNPs, air, and gel-like photopolymer. Gel-like photopolymer is visible behind the agglomerations (Figure 8B, above agglomeration, and Figure 8D, below agglomeration), which blocked the UV light from passing through them. Such phenomena are not observed in other cross-sections reported here. No voids or porosity apart agglomerations are present.

Overall, looking at the results, it is clear that the use of ultrasonic bath and stirring does not provide enough energy to disperse nanomaterials into photopolymer matrix, independent of the solvent used. The best dispersion is achieved in MWFE composites, however, some agglomerations are still present, and the clusters of dispersed MWCNTs are visible. Therefore, it is expected that ethanol might provide better dispersion with other nanomaterials as well, especially if higher energy tools are used, such as ultrasonic sonotrode. The partial dispersion in MWFE also explains the rough surface of the specimen, where the clusters of partially dispersed MWCNTs blocked the UV light and caused fluctuations of light intensity over the cross-section.

### 3.2. Cure Depth

**Figure 9:**
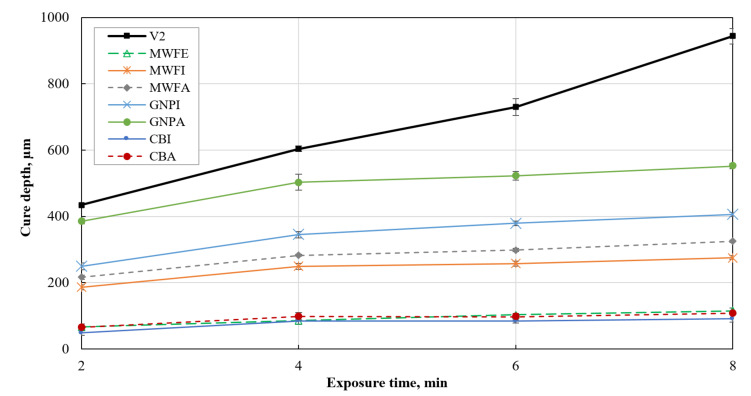
Cure depth of the suspensions at different exposure times.

Cure depth is the most important parameter that defines material printability, since it defines how deep the UV light can penetrate and solidify the structure over a specific time. While conducting a cure depth experiment, it became particularly apparent that cure depth is highly dependent on nanomaterial and solvent used. Figure 9 shows the cure depth of all suspensions made, including the cure depth of pure photopolymer resin as purchased.

The specimen thickness made of pristine photopolymer increases from 435 µm (at 2 min) up to almost 1 mm (at 8 min), which corresponds to the height of the mold used in the experiment. The increase in cure depth with an increase of UV light exposure time is expected. All the composite specimens have a reduced thickness compared to the pristine photopolymer, irrespective of the solvent used. All suspensions show the sharpest increase in cure depth when exposure time from 2 to 4 min is used, and then the cure depth starts to increase slower. A similar trend of results is reported in the literature for composites made with MWCNTs [32].

The highest cure depth is reached with the GNPA suspension, and the lowest cure depths are achieved with CBI, CBA, and MWFE suspensions. In the case of MWFE, the low cure depth can be easily explained by the partial dispersion of MWCNTs. Improved nanomaterial dispersion increases UV light scattering and blocks more UV light, thus strongly decreasing cure depth.

In the case of CBA and CBI, many small agglomerations are present in the solidified specimens, thus the cure depth should be similar to other composites manufactured in this study (except MWFE). The extremely low cure depth of CBA and CBI composites can be explained by the huge UV absorption of CB particles. CB is a well-known UV absorber and helps to increase the UV protection of various polymers and epoxies [47,48,49,50]. Therefore, extremely low cure depth indeed shows the non-suitability of CB to be a dielectric filler for UV light-based 3D printable composites.

On the other hand, MWFI and MWFA produced average cure depth results, mostly because MWCNTs themselves only start to absorb most of UV light starting with wavelengths smaller than around 380 nm [51], while in this study, UV light source with a wavelength of 395 nm is used. Furthermore, only huge agglomerations formed inside, thus they block UV light only locally. Although MWFA showed some minor agglomerations inside, it still produced a higher cure depth than MWFI, which shows no minor agglomerations. Some small agglomerations in MWFI that were not found during SEM imaging might be a possible explanation.

Both composites with GNPs produced the highest cure depth of investigated composites. Looking at the SEM images of GNPA and GNPI (Figure 8), it is clear that only huge agglomerations are present. Therefore, only local disturbance in UV light exposure happens, which does not block most of the UV light, and thus allows a deeper cure. GNPA produced higher cure depth than GNPI, most likely because of fewer agglomerations overall, although they are slightly bigger.

In literature, it is reported that GNPs also absorb UV light, however in the range of 100-320 nm, while they scatter the UV light in higher wavelengths [50]. The scattering of light instead of absorption could help to explain the high cure depth of GNPI and GNPA. GNPs also have a huge specific surface area, which acts as a physical barrier to UV light [50], but GNPs used in this study seemed to be fragmented, and this might produce less UV light blocking than higher-quality GNPs. Furthermore, their dispersion is extremely poor in suspensions prepared in this study, and physical blocking phenomena most likely are not observable.

The different cure depth of the composites is attributed to the nanomaterial geometry, its dispersion, and distribution. The homogenous dispersion and distribution of nanomaterials in the composite result in stronger absorption of the UV light, which in turn, significantly reduces the cure depth of the suspensions [32,52]. This is obvious when comparing MWFE with other composites prepared in this study.

Different geometry of the nanofillers might also influence cure depth differently. It is likely that the low content of the GNPs does not affect the curing depth to the same extent as with the other nanomaterials, especially at poor GNPs dispersion, as reported in this study. Feng [43] reported a thickness reduction of 70% compared to pure resin when adding 0.5 wt.% GNPs. In contrast, in this study, the GNP content is five times smaller, and the GNPs are also agglomerated. As a result, the reduction only ranges between 16% (4 min) and 28% (6 min). A comparison with the work of Eng et al. [32] indicates that the light source, resin, and composite preparation process can lead to different cure depth results as well. While in this work, the curing depth of the MWFE dispersed with a help of ethanol is only around 66 µm (2 min), Eng et al. report a depth of approximately 250 µm, although they used 0.25 wt.% MWCNTs, and achieved better dispersion [32]. However, they do not provide any information about the distance to the UV light source, which is a crucial factor determining the UV light intensity at the surface of the suspension.

Considering the solvent used, all composites dispersed with the help of acetone produced a slightly higher cure depth (especially GNPs) than the composites prepared with isopropyl alcohol. It cannot be fully concluded, but it seems that isopropyl alcohol is more suitable for nanomaterial dispersion than acetone is.

Concluding cure depth results, it is clear that cure depth is dispersion quality dependent, which in turn, depends on the nanomaterial, solvent, and dispersion method/devices used. Good dispersion of nanomaterials reduces cure depth, even at very low amounts, such as 0.1 wt.%, where huge agglomerations have a lower influence to cure depth. However, some nanomaterials, such as CB, seem to block/absorb a lot of UV light, even with huge agglomerations, which in turn, would produce unprintable composites in the future, with better dispersion of CB. Both MWCNTs and GNPs look promising for further investigation.

### 3.3. Dielectric Properties (as Cured Specimens)

**Figure 10:**
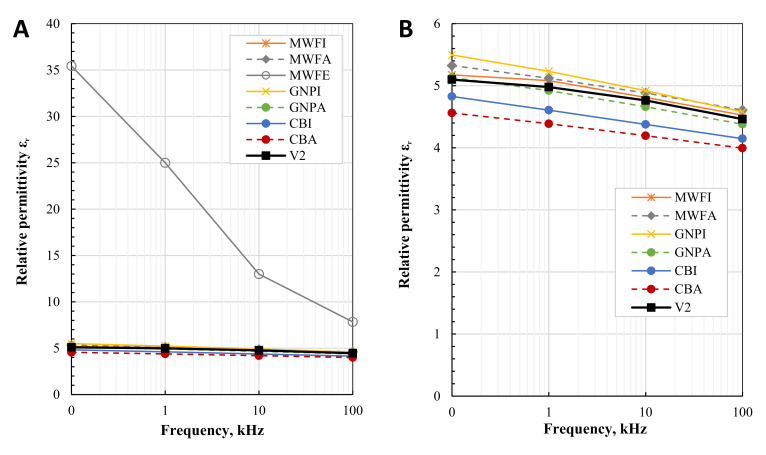
Relative permittivity ε_r_ of: (**A**) all composites; (**B**) composites without MWFE.

**Figure 11:**
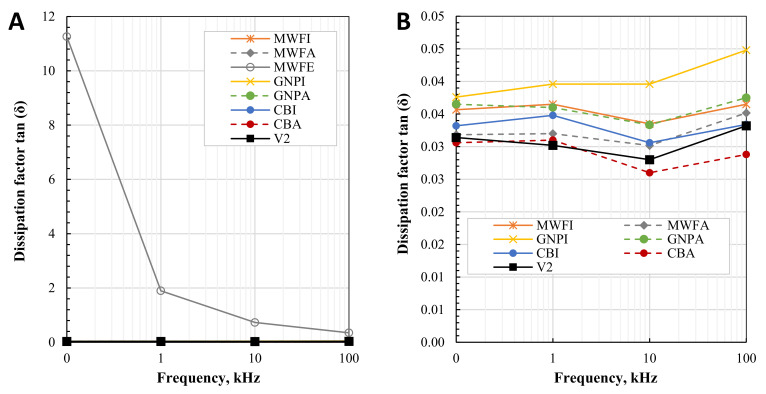
Dissipation factor tan (δ) of: (**A**) all composites; (**B**) composites without MWFE.

The relative permittivity ε_r_ and dissipation factor tan (δ) characterize the dielectric properties of the composites. For the optimum performance of piezoelectric composites, planned to be 3D printed in the future, an increase in relative permittivity is desired without a large increase in dielectric losses, indicated by a high dissipation factor. Figure 10 shows the relative permittivity ε_r_ of the pure resin and 0.1 wt.% nanomaterial composites, over the frequency range from 100 Hz up to 100 kHz. Figure 11 shows the dissipation factor tan (δ) over the frequency range from 100 Hz up to 100 kHz.

Relative permittivity decreases as the frequency of all composites increases, as expected. Regarding the SEM images, ethanol led to the most homogenous dispersion of the nanotubes. As a consequence, a conductive network forms in MWFE even at 0.1 wt.%, leading to an increase of ɛ_r_ up to 35.43 (at 0.1 kHz), and a strong drop over the frequency to 7.84 (at 100 kHz). The same applies to the dissipation factor tan (δ) (see Figure 11), which decreases from 11.26 (at 0.1 kHz) to 0.35 (at 100 kHz), indicating the occurrence of leakage current [22,53]. This behavior is expected with the addition of nanomaterials at percolation. The dissipation factor of the other composites varies very little, and of all composites (except MWFE) and pure photopolymers, is between 0.03 and 0.04, thus 0.1 wt.% of poorly dispersed nanomaterials in the photopolymer shows a very small increase in dissipation factor tan (δ).

Almost all composites prepared show slightly increased relative permittivity, where GNPI shows increased ε_r_ up to 18% (at 1 kHz). However, GNPA produced no increase in ε_r_ at low frequencies compared to pure photopolymer, and even produced slightly lower ε_r_, at higher frequencies than the photopolymer itself produces. The low dielectric increase of GNP composites can be attributed to extremely poor dispersion and the low nanomaterial content. No significant changes in conductivity are reported in the literature for composites containing up to 0.5 wt.% of GNPs [28]. Due to their morphology, higher weight fractions of up to 4–6 wt.% [21] or even up to 15 wt.% can be necessary, depending on their lateral size, whereas MWCNTs or CB can produce higher dielectric properties at much lower concentrations [26]. Both MWCNTs composites increased ε_r_ only slightly. Both CB composites decreased ε_r_ up to 12% (at 1 kHz). A decrease in permittivity can be caused by chemically compounded surfaces or material defects and impurities [54].

### 3.4. Dielectric Properties (after Post-Curing)

Photopolymers solidify under UV light, where the degree of the cure depends on the wavelength of light used, distance to the object, and exposure time used. The precise combination of these parameters defines the final properties of cured material. The photopolymer used in this study requires a subsequent post-curing step after the printing step to achieve final mechanical properties and high-temperature resistance. It is believed that the degree of cure should also influence dielectric properties. However, the manufacturer of the photopolymer does not provide any data.

The material data of the polymer provided by the manufacturer are applicable only when the material is used with a respective 3D printer that uses a UV laser. However, in this work, a customized LED UV light source, made of LED light strips, as presented earlier, is used. Furthermore, the addition of nanofillers also hinders the curability of the suspensions. Therefore, the exact degree of the cure of the suspensions is unknown, and no data from the manufacturer can be applied.

Additional post-curing is performed on the composites to determine their influence on the dielectric properties. The same specimens already with gold electrodes, as used for the initial measurement of dielectric properties, are placed between two thin glass plates, and are placed inside the post-curing device (Form Cure, Formlabs, USA) for 10 min. The glass plates ensure specimen flatness during post-curing. The post-curing device has 13 multi-directional LEDs (wavelength 405 nm) with a total LED power of 39 W (LED radiant power is 9.1 W) [55]. Sputtered electrodes cover a large area of the specimen from both sides, and might strongly influence the post-curing, especially curing introduced by UV light. The heating function is activated in a post-curing device to maintain 60 °C, as defined by post-curing parameters by the manufacturer of a photopolymer. After 10 min of post-curing, specimens are taken out, and their dielectric properties are measured.

**Figure 12:**
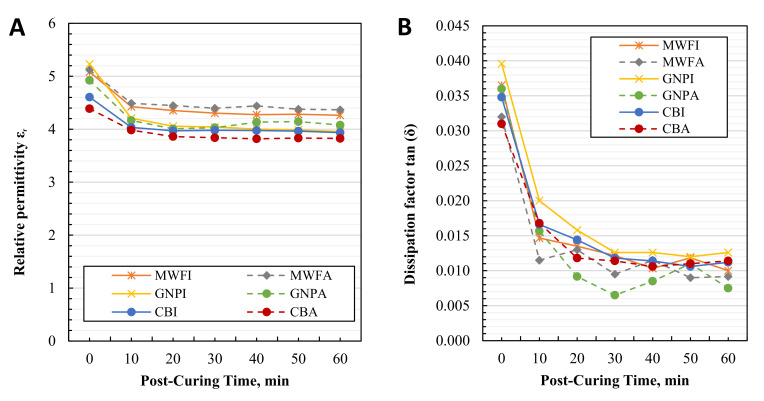
Results of post-cured 0.1 wt.% nanomaterial/photopolymer composite specimens: (**A**) change in relative permittivity ε_r_ at 1 kHz; (**B**) change in dissipation factor tan (δ) at 1 kHz.

Figure 12A shows the change in the relative permittivity ε_r_ at 1 kHz of the composites after every 10 min of post-curing, while Figure 12B shows the change in dissipation factor tan (δ) at 1 kHz. Other frequencies are not shown, because the resulting trend is similar, as shown in Figure 10.

Results after post-curing show a sharp decrease in relative permittivity ε_r_ after post-curing for 10 min for all specimens. The decrease is, on average, approx. 12%. Further post-curing slightly decreases relative permittivity ε_r_ on average another 3%. The only variance can be noticed with GNPA and MWFA, and very small with CBA, where relative permittivity ε_r_ slightly increased after 40 min of post-curing and started to drop again. The exact reason for this behavior is unknown at the moment, but most likely, it is a measurement error. These composites are dispersed with the help of acetone. Composites dispersed with isopropyl alcohol show no such behavior. A decrease in electrical properties with an increase in a degree of cure is reported in the literature for 0.5 wt.% of GNPs composites, where the results of ε_r_ are related to the immobility of charges as a degree of cure increases [28].

Figure 12B shows the change in dissipation factor tan (δ) over post-curing time. The same trend as for relative permittivity is followed. However, the decrease is much higher, and in some cases, the decrease in dissipation factor is up to 300%.

Two-component composites manufactured in this study are the first step for three-component piezoelectric composites manufacturing. By knowing the influence of post-curing on dielectric properties, the most appropriate step in manufacturing can be used to polarize 3D printed piezoelectric composites, due to the highest relative permittivity ε_r_, which is desirable. Current results suggest that the specimens with a low degree of cure are the best for polarization, because of the highest relative permittivity ε_r_, which decreases with additional post-curing. However, post-curing is required to achieve the final mechanical properties of the composite, and thus higher piezoelectric output of piezoelectric composites due to the stiffer polymer matrix is possible [9].

### 3.5. Viscosity of Nanocomposites

**Figure 13:**
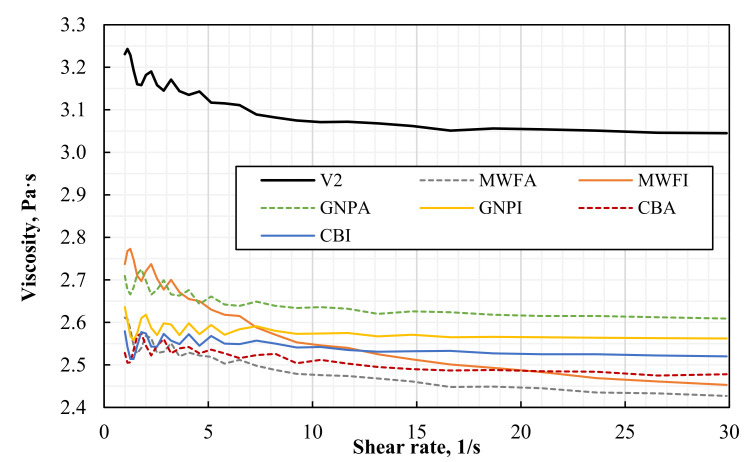
Viscosities of 0.1 wt.% nanomaterial/photopolymer composites and pristine “High-Temperature V2” photopolymer resin at shear rates from 1 to 30 s^−1^.

The viscosity of the suspensions used in 3D printing must be low to achieve self-leveling, and at the same time, thin printable layers must be formable. To understand the influence of nanomaterials on the viscosity of the suspensions, the viscosities of all suspensions prepared are measured. Figure 13 shows the viscosity over a varying shear rate. The viscosity of the pure “High-Temperature V2” photopolymer resin without any nanofillers is taken as a reference. MWFE suspension was not measured because of poor early results.

Very interestingly, the viscosities of all composites are reduced compared with a pure photopolymer. Although the reasons for this behavior are not yet fully understood, it can be suggested that the addition of solvent and the following evaporation might influence the rheological properties of the photopolymer resin itself. Multiple measurements of pure photopolymer on two different devices have been conducted to exclude any error.

The manufacturer specifies the viscosity of photopolymer as 1.015 Pa·s at 35 °C. Since the measurement in this work is performed at a temperature of 22 °C, the viscosity is above 3 Pa·s. Photopolymer viscosities of similar works [24,43] were not higher than 0.5 Pa·s at room temperature, which classifies “High-Temperature V2” photopolymer as a higher viscosity photopolymer. However, the increased viscosity at room temperatures is a very advantageous characteristic, because its high viscosity reduces filler sedimentation. To achieve a printable low-viscosity suspension, it can be slightly heated in the printer.

Figure 13 indicates the decrease in viscosity for MWFI, MWFA, and GNPA suspensions, with increasing shear rate up to approximately 10 s^−1^. Composites with MWCNTs show continuously decreasing viscosity, even at shear rates higher than 10 s^−1^. Since the MWCNT dispersions show the strongest decreases in viscosity, it is expected that they contain larger agglomerations or smaller particle interspacing than the other nanomaterial suspensions. As a result, the flow of the resin is blocked. The viscosity of GNPA and CB mixtures is almost constant over varying shear rates. Possibly, the viscosity starts to increase at a higher weight fraction, as observed by León and co-workers for GNPs [28]. Overall, the change in viscosity of suspensions with 0.1 wt.% nanomaterials is comparably small and almost shear-rate independent at higher shear rates. Poor dispersion of nanomaterials is still able to influence the viscosity of the suspensions.

In the past, it has been shown that viscosity is increased by the addition of nanomaterials. Even small amounts, such as 0.1 wt.% MWCNTs, can cause a viscosity increase by 2.7 Pa·s at 1 s^−1^ [23]; however, no solvent is used in their work during dispersion. Mu et al. [24] determined a maximum weight fraction of 0.3 wt.% MWCNTs for optimum printability, where higher loading increases viscosity higher. In contrast, with 0.5 wt.% GNPs and a viscosity of 0.742 Pa·s, the printability was not limited in the work of Feng [43]. This is in accordance with León and Molina [28], where a maximum 3D printing suitable weight fraction of 2.5 wt.% for GNPs has been determined. Moreover, an increase in viscosity became apparent from 1 wt.% GNPs/Form Clear V2 resin (0.919 Pa·s (0%) to 1.13 Pa·s) only [28]. Therefore, similar observations were expected in this study.

## 4. Conclusions

In summary, 3D printable, two-component 0-3 composites, consisting of 0.1 wt.% carbon-based nanomaterials in photopolymer were prepared using an ultrasonic bath and magnetic stirrer. Nanomaterial influence over cure depth, dielectric properties, and viscosity was systematically investigated. SEM images revealed the poor dispersion of nanomaterials. The dispersion quality of the solvents (acetone and isopropyl alcohol) showed no significant differences in microstructure, cure depth, viscosity, and dielectric properties.

The poor dispersion of nanomaterials is attributed to the insufficient power of the ultrasonic bath used in the dispersion process. The use of an ultrasonic sonicator is suggested to improve the dispersion of nanomaterials. Regardless of poor dispersion, the cure depth experiences a great reduction via the addition of nanomaterial. The highest cure depth is achieved with GNPs.

Because of the rather low nanomaterial content, all composites (except MWFE) showed no significant increase in dielectric properties. The highest increase in relative permittivity up to 18% (at 1 kHz) is observed in the GNPI composite. In contrast, CB decreased relative permittivity by up to 12% (at 1 kHz). No changes in dissipation factor tan (δ) are observed, except for MWFE composites, where the dissipation factor decreases with frequency (composite is at, or above, percolation threshold).

Subsequent post-curing reveals a slight decrease in relative permittivity and a high decrease in dissipation factor with an increase of post-cure time.

To summarize, an ultrasonic bath cannot provide sufficient energy to disperse nanomaterials into the photopolymer. While CB performed worse in our study because of huge UV light absorption, the use of MWCNTs and GNPs together with ethanol as a dispersant is recommended for further studies in this direction.

## Data Availability

Not available.
